# Human Papillomavirus Vaccination and Human Papillomavirus–Related Cancer Rates

**DOI:** 10.1001/jamanetworkopen.2024.31807

**Published:** 2024-09-05

**Authors:** Victor Adekanmbi, Itunu Sokale, Fangjian Guo, Jessica Ngo, Thao N. Hoang, Christine D. Hsu, Abiodun Oluyomi, Abbey B. Berenson

**Affiliations:** 1 Center for Interdisciplinary Research in Women’s Health, School of Medicine, The University of Texas Medical Branch, Galveston; 2 Department of Obstetrics and Gynecology, The University of Texas Medical Branch, Galveston; 3 Department of Medicine, Section of Epidemiology and Population Sciences, Baylor College of Medicine, Houston; 4 Dan L. Duncan Comprehensive Cancer Center, Baylor College of Medicine, Houston, Texas; 5 School of Medicine, The University of Texas Medical Branch, Galveston; 6 Institute for Translational Sciences, The University of Texas Medical Branch, Galveston

## Abstract

**Importance:**

To inform the design and implementation of targeted interventions to reduce the future burden of human papillomavirus (HPV)–related cancers in Texas, it is necessary to examine the county and health service region (HSR) levels of (1) the proportion of children and teenagers aged 9 to 17 years who initiated and were up to date for HPV vaccination series and (2) HPV-related cancer incidence rates (IRs).

**Objective:**

To evaluate temporal trends and geospatial patterns of HPV vaccination initiation and up-to-date status as well as HPV-related cancer rates at county and HSR levels in Texas.

**Design, Setting, and Participants:**

This population-based cross-sectional study used data from the Texas Immunization Registry, the National Cancer Institute’s Surveillance, Epidemiology, and End Results Program database, and Texas Department of State Health Services annual population counts from 2006 to 2022. The analysis of HPV vaccination rates was conducted among children and teenagers aged 9 to 17 years; the analysis of HPV-related cancer rates was conducted among adults aged 20 years and older. Data were extracted between June and July 2023 and statistical analysis was performed from February to April 2024.

**Main Outcomes and Measures:**

HPV vaccination initiation and up-to-date status rates and HPV-related cancer IR at county and HSR levels.

**Results:**

A total of 32 270 243 children and teenagers (65.8% female individuals and 34.2% male individuals) and 22 490 105 individuals aged 20 years and older (50.7% female individuals and 49.3% male individuals) were included. The mean 2021 to 2022 county-level HPV vaccination series initiation estimates ranged from 6.3% to 69.1% for female and from 7.0% to 77.6% for male children and teenagers aged 9 to 17 years. County-level vaccination up-to-date estimates were generally lower compared with those of initiation estimates and ranged from 1.6% to 30.4% for female and from 2.1% to 34.8% for male children and teenagers. The pattern of HPV vaccination rates stratified by sex were similar across counties and HSRs. The age-adjusted annual HPV-related cancer IR by county for years 2016 to 2020 ranged from 0 to 154.2 per 100 000 for female individuals and from 0 to 60.1 per 100 000 for male individuals. The counties located in North Texas, HSRs 2/3 and 4/5N, had lower HPV vaccination rates and higher IRs of HPV-related cancers for both female and male individuals compared with other regions.

**Conclusions and Relevance:**

In this study, the incidence of HPV-related cancers varied widely across the counties and HSRs of Texas. More counties in North Texas, HSRs 2/3 and 4/5N, had higher IRs of HPV-related cancers and a lower proportion of HPV vaccination rates than counties in other regions. Designing and implementing targeted interventions to increase uptake and completion of HPV vaccination series across counties with low HPV vaccination rates may help to reduce future the burden of HPV-related cancers.

## Key Points

**Question:**

Are there disparities in human papillomavirus (HPV) vaccination initiation and up-to-date rates and HPV-related cancer incidence across counties and health service regions (HSRs) in Texas?

**Findings:**

In this cross-sectional study with more than 54.7 million participants, county- and HSR-level HPV vaccination series initiation rates and up-to-date status as well as the incidence of HPV-related cancer varied widely. The counties in the northern region of Texas had a higher incidence of HPV-related cancers and lower HPV vaccination rates compared with those in other regions.

**Meaning:**

The finding that North Texas had lower HPV vaccination series initiation and up-to-date status with a higher HPV-related cancer burden than other regions raises concern, as this may indicate continued widening of existing health disparities between North Texas and other regions.

## Teaser

This cross-sectional study examines human papillomavirus (HPV) vaccination rates and the incidence of HPV-related cancer at the county level in Texas.

## Introduction

The United States has a high human papillomavirus (HPV) disease burden, with more than 42 million people infected with disease-associated HPV types and 13 million people acquiring a new infection in 2018.^1^ Additionally, approximately 30 700 HPV-attributable cancer cases are newly diagnosed in the United States each year.^2^ With the introduction of the HPV vaccine in 2006, HPV-associated infections, warts (HPV types 6 and 11), and cancers (HPV types 16 and 18) became preventable.^3^ The HPV 9-valent vaccine that is now available for use in the United States protects against 9 high-risk (HR) HPV types (6, 11, 16, 18, 31, 33, 45, 52, and 58) and is recommended as a 2-dose series for individuals aged 9 to 14 years or a 3-dose series for ages 15 to 26 years.^4^ The HPV vaccination series has been found to be highly effective, with efficacy rates close to 100% in preventing the development of HPV-associated cancers among individuals without prior infection of the HPV vaccine types.^5^ Despite its safety and high efficacy, national HPV vaccination rates are well below the Healthy People 2030 goal of 80% coverage.^6^

Compared with other US regions, Southern states, including Texas, have lower HPV vaccination series initiation, up-to-date, and completion rates and higher incidence rates (IRs) of cervical cancer, a well-known HPV-related cancer.^7,8^ In female individuals in Texas, an estimated 83% of HPV-related cancer incidence is attributable to HR-HPV infection, while in male individuals, the estimate is 74%,^8^ indicating a need for increased HPV vaccination initiation, completion, and coverage as well as HPV-related cancer surveillance. According to the National Immunization Survey–Teen in 2022, the estimated HPV vaccination coverage among teenagers aged 13 to 17 years in Texas is 58.5%, which is below the national estimate of 62.6% for the same age group.^9^ Texas ranks 48th in HPV vaccination series completion and 44th in HPV vaccination series initiation (≥1 dose) among US states for eligible adolescents and young adults,^10,11^ highlighting the state’s low HPV vaccination rates. Available statistics indicate that the total population estimates of individuals aged 12 to 17 years in Texas for year 2022 was 2 635 339 compared with 1 525 176 for Florida, 1 378 121 for New York, 937 432 for Pennsylvania, and 25 810 168 for the rest of the United States,^12^ which makes it important to raise HPV vaccination coverage rate among this age group in Texas.

Disparities in HPV vaccination and HPV-related cancer incidence also exist within areas of Texas. Between 2013 and 2017, 3200 incident cases of HPV-related cancers were diagnosed annually, with higher IRs in rural than urban regions (13.0 vs 11.7 per 100 000, respectively).^8^ HPV vaccination coverage in Texas regions also varied among teenagers aged 13 to 17 years in 2017, with percentages of vaccine initiation ranging from 53% to 85% and up-to-date vaccination ranging from 30% to 51% across 6 distinct regions.^13^ Furthermore, it is important to examine HPV vaccination rates by sex (female and male) as a result of the significant differences in HPV-related cancer rates between female and male individuals and because the introduction of HPV vaccination for male patients came 5 years later after that for female patients.^14,15,16,17^

Given the HPV vaccination disparities within Texas as well as the low rates of vaccination coverage in Texas compared with the national estimates,^13^ we sought to examine the Texas county- and health services region (HSR)–specific proportion of children and teenagers aged 9 to 17 years who initiated and were up to date for HPV vaccination series and to examine the HPV-related cancer IR among adults. The aims of this study were to provide county-specific data for Texas on HPV vaccination uptake and up-to-date status and HPV-related cancer incidence to inform targeted interventions.

## Methods

### Study Population

Our study population consists of (1) children and teenagers aged 9 to 17 years who received at least 1 dose (initiated) and were up to date (all vaccine recommended doses) for the HPV vaccination series and (2) HPV-related cancer IR among adults aged 20 years and older across Texas. The state of Texas has 254 counties, which is the highest number for any US state. Texas Department of State Health Services (DSHS) grouped the 254 counties in the state into 11 Public Health Regions served by 8 regional offices, which are HSR 1, HSR 2/3, HSR 4/5N, HSR 6/5S, HSR 7, HSR 8, HSR 9/10, and HSR 11 (eFigure in Supplement 1). The roles of the HSRs include the organizing and provision of comprehensive public health services to Texas residents.^18^ Other roles of HSRs of Texas are resource sharing, provision of direct support to county and local public health agencies, and leadership and coordination of public health emergency preparedness and response. Each county is assigned to 1 of the 8 HSRs for administrative purposes. HSR 1, with 41 counties, has the highest number of counties, and HSR 11, with 19 counties, has the lowest number of counties.^19^ The University of Texas Medical Branch institutional review board reviewed this study and classified it as exempt because it used publicly available deidentified county- and state-level data. This report follows the Strengthening the Reporting of Observational Studies in Epidemiology (STROBE) reporting guideline.

### Data Requirements and Measures

Datasets used included (1) Texas Immunization Registry (2006-2022), (2) Texas HPV-related cancer incidence from the National Cancer Institute’s Surveillance, Epidemiology, and End Results Program database (2016-2020), and (3) Texas DSHS for yearly population counts (2006-2022). The total number of HPV vaccinations administered by county, HSR, sex, year (2006-2022 for female and 2011-2022 for male individuals), and ages when received first and subsequent doses (for up-to-date status) were requested from the Texas Immunization Registry. Definitions of HPV-related cancer used were based on the following: (1) oropharyngeal cancer, *International Classification of Disease for Oncology*, *Third Edition* (*ICD-O-3*)^20^ site codes C01.9, C02.4, C02.8, C05.1, C05.2, C09.0, C09.1, C09.8, C09.9, C10.0 to C10.4, C10.8, C10.9, C14.0, C14.2, and C14.8, with *ICD-O-3* histology codes 8050 to 8086 and 8120 to 8131; (2) anal and rectal cancers, *ICD-O-3* site codes C20.9, C21.0 to C21.2, and C21.8, with *ICD-O-3* histology codes 8050 to 8084 and 8120 to 8131; (3) vulvar cancer, *ICD-O-3* site codes C51.0 to C51.2, C51.8, and C51.9, with *ICD-O-3* histology codes 8050 to 8084 and 8120 to 8131; (4) vaginal cancer, *ICD-O-3* site code C52.9, with *ICD-O-3* histology codes 8050 to 8084 and 8120 to 8131; and (5) cervical cancer *ICD-O-3* site codes C53.0, C53.1, C53.8, and C53.9, with *ICD-O-3* histology codes 8010 to 8671 and 8940 to 8941.

### Statistical Analysis

#### Calculation of HPV Vaccination Coverage

We calculated HPV vaccination coverage with cumulative number of children and teenagers aged 9 to 17 years who initiated or were up-to-date as the numerator for the rate and the annual total population of children and teenagers aged 9 to 17 years from the DSHS as the denominator. Based on the years that Centers for Disease Control and Prevention’s Advisory Council on Immunization Practices recommended the HPV vaccine universally, HPV vaccination rates by county and HSR region were calculated for female individuals from 2006 to 2022 and for male individuals from 2011 to 2022. Excel version XX (Microsoft) was used to carry out the temporal trend analyses.

#### Calculation of HPV-Related Cancer IRs

We used the direct method^21^ to estimate the mean yearly age-adjusted HPV-related cancer IRs. Multiple diagnoses of the same HPV-related cancer for any patient in a single year period were used as only 1 diagnosis. We applied the mean annual HPV-related cancer IR derived from years 2016 to 2020 to the US 2000 standard population weights to obtain our mean annual age-adjusted cancer IRs.^22^ In addition, we carried out a Spearmen correlation matrix between HPV vaccination initiation, HPV vaccination up-to-date status, and HPV-related cancer incidence across all counties, separately by sex.

#### Geospatial Variation

For assessment of geospatial patterns of the HPV metrics of interest through univariate mapping, HPV vaccination series initiation, HPV vaccination up-to-date status, and HPV-related cancer rates were divided into terciles. Each tercile was color coded with increasingly darker colors indicating increased incidence of HPV-related cancers.^14^ Bivariate choropleth maps were thereafter generated to show concurrent county-level HPV vaccine initiation prevalence and HPV-related cancer IR for each sex, in which 3 terciles of HPV vaccine initiation and 3 terciles of HPV-related cancer incidence were separated into 9 values to allow for greater variability in the values of displayed variables.^14,23^ All geospatial analyses were done in ArcGIS Pro software version 3.2 (Esri).

## Results

**Figure 1.**
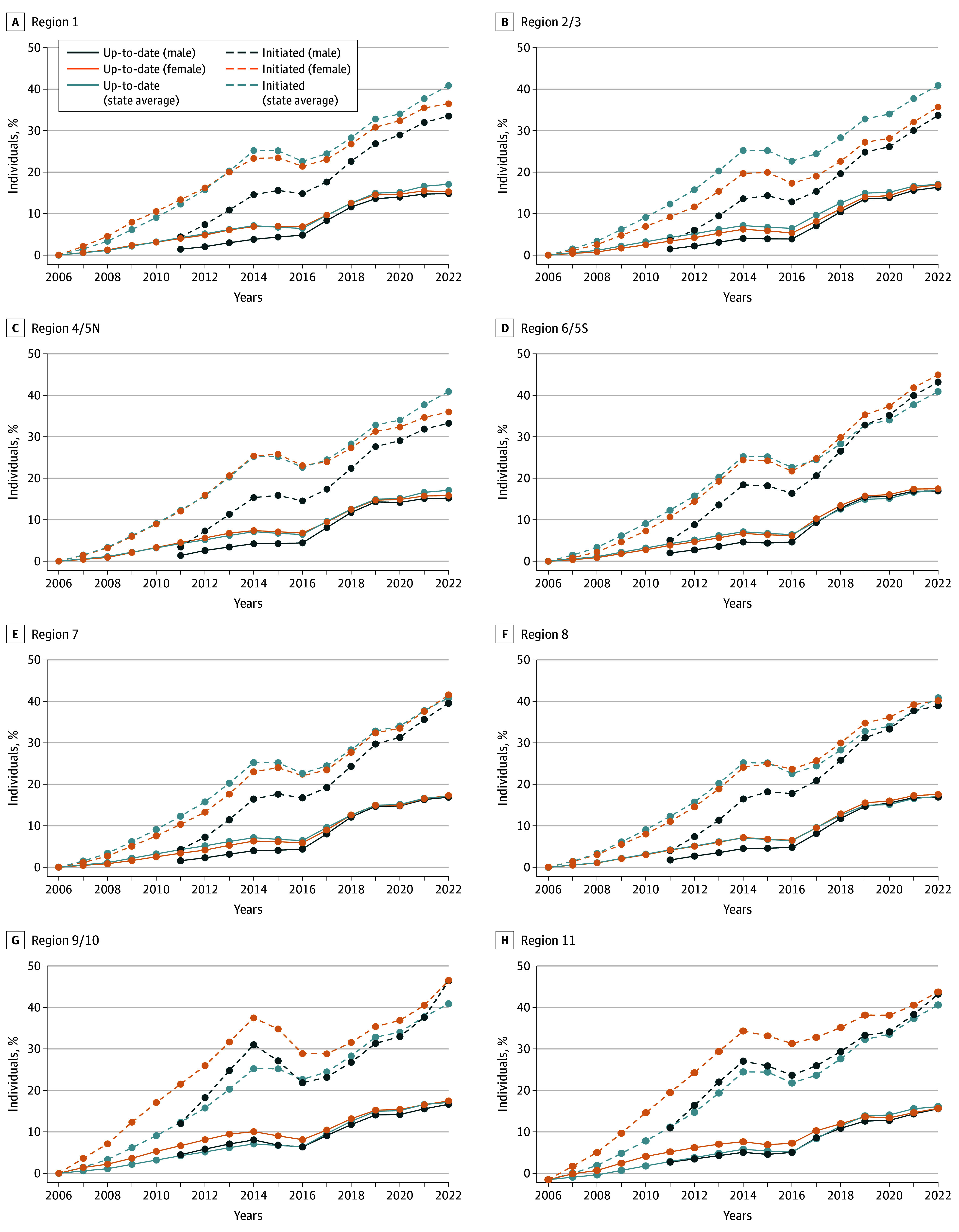
Human Papillomavirus Vaccine Initiation and Up-to-Date Estimates Among Female and Male Individuals From 2006 to 2022, by Texas Health Service Region

A total of 32 270 243 children and teenagers (65.8% female individuals and 34.2% male individuals) and 22 490 105 individuals aged 20 years and older (50.7% female individuals and 49.3% male individuals) were included. HPV vaccination series initiation among children and teenagers aged 9 to 17 years in the state of Texas increased among female individuals from 0% in 2006 to 40.9% in 2022 (Figure 1). HPV vaccination initiation among male individuals increased from 5.8% in 2011 to 39.3% in 2022 (Figure 1). The up-to-date HPV vaccination status also increased for female participants from 0% in 2006 to 17.1% in 2022 and from 2.2% to 16.6% for male participants.

**Figure 2.**
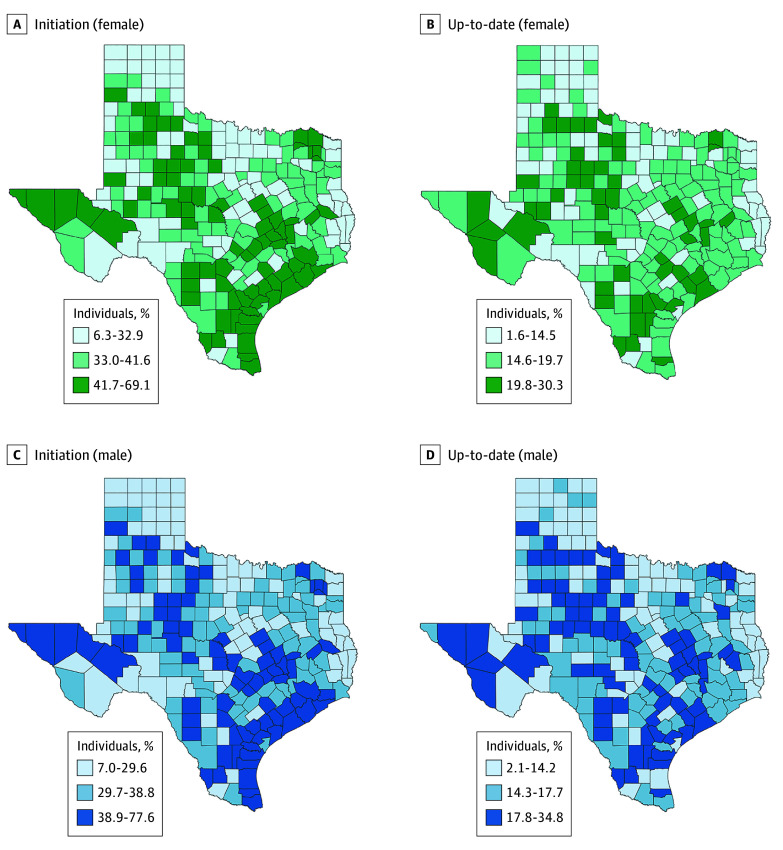
Human Papillomavirus Vaccine Initiation and Up-to-Date Estimates Among Female and Male Individuals From 2021 to 2022, by County

The mean county-level HPV vaccination initiation rates for 2021 to 2022 ranged from 6.3% to 69.1% for females (62.8–percent point spread) and from 7.0% to 77.6% for males (70.6–percent point spread) (Figure 2). More counties and HSRs in North Texas were found to have HPV vaccination initiation and up-to-date estimates in the lowest tercile for both female (6.3%-32.9%) and male (7.0%-29.6%) individuals compared with other regions.

In Texas in 2022, 16.6% of male individuals and 17.1% of female individuals aged 9 to 17 years were up to date with their HPV vaccination series. Across all the counties studied, the mean 2021 and 2022 proportion of children and teenagers aged 9 to 17 years old who had up-to-date status for HPV vaccination series ranged from 2.1% to 34.8% for male participants and from 1.6% to 30.4% for female participants. Most of the counties with up-to-date HPV vaccination status in the lowest tercile for both male (2.1%-14.2%) and female (1.6%-14.5%) individuals were in North Texas (Figure 2).

**Figure 3.**
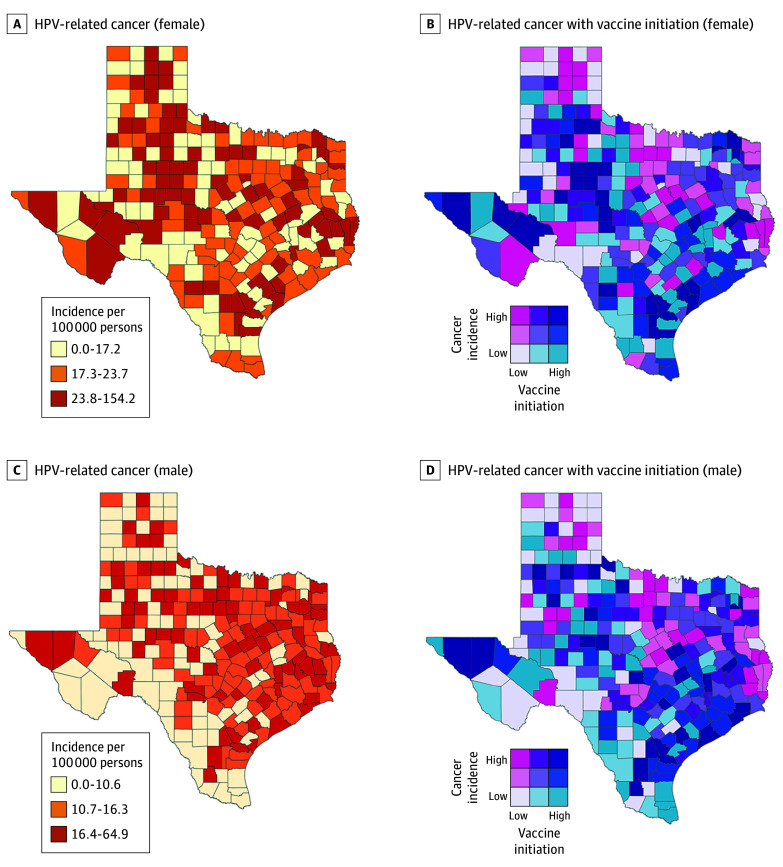
Human Papillomavirus (HPV)–Related Cancer Incidence Rate Alone and With HPV Vaccination Initiation Among Female and Male Individuals From 2021 to 2022

The Texas yearly mean age-adjusted HPV-related cancer IR for years 2016 to 2020 was 22.1 per 100 000 female individuals and 14.3 per 100 000 male individuals. The county-level HPV-related cancer IR ranged from 0 to 154.2 per 100 000 for female individuals and 0 to 64.9 per 100 000 for male individuals (Figure 3). Most counties that had HPV-related cancer IRs within the highest tercile for female individuals (23.8 to 154.2 per 100 000) were in North Texas. Similarly, most counties with the highest tercile of HPV-related cancer IRs for male individuals (16.4 to 64.9 per 100 000) were in North Texas (Figure 3). Complete results of county-level HPV vaccination initiation and up-to-date estimates as well as HPV-related cancer IRs are available online in eTable 1 in Supplement 1 for female individuals and eTable 2 in Supplement 1 for male individuals.

For HSRs, HSR 2/3 (35.7% and 33.7%) and HSR 4/5N (36.0% and 33.3%) had lower HPV vaccination initiation rates for both female and male children and teenagers compared with other HSRs (Figure 1). HSR 9/10 (46.5% and 46.4%) and HSR 11 (43.9% and 43.5%) had higher HPV vaccination rate for both female and male children and teenagers compared with other HSRs as well as the state average rate (40.9%) (Figure 1).

**Figure 4.**
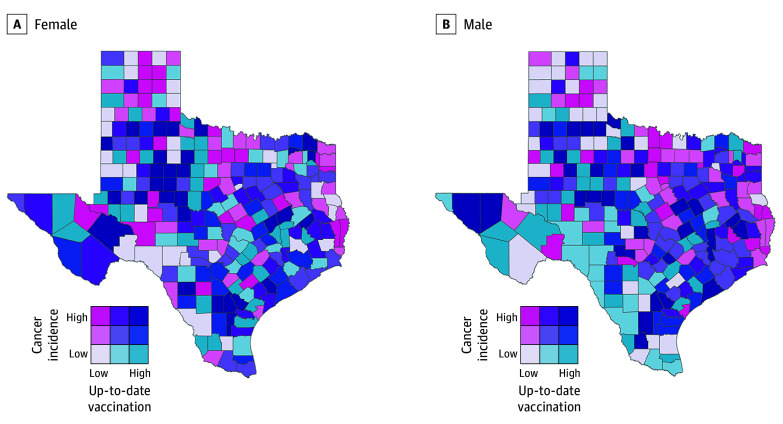
Human Papillomavirus–Related Cancer Incidence (2016-2020) and Human Papillomavirus Vaccination Up-to-Date Status Among Female and Male Individuals From 2021 to 2022

The HPV-related cancer IRs as well as HPV vaccination initiation rate across the counties were examined concurrently in bivariate choropleth maps (Figure 3). The counties color coded hot pink had the highest rate of HPV-related cancer and the lowest level of vaccine initiation, while counties coded dark purple had the highest rates of vaccination and the lowest rates of HPV-related cancer. South Texas had the lowest incidence of HPV-related cancers for both female (≤17.2 per 100 000) and male (≤10.6 per 100 000) individuals and the most counties with high HPV vaccination initiation (≥41.7% for female individuals; ≥38.9% for male individuals) and up-to-date rates (≥19.8% for female individuals; ≥17.8% for male individuals) (Figure 3 and Figure 4). Complete results of a Spearmen correlation matrix between HPV vaccination initiation, HPV vaccination up-to-date status, and HPV-related cancer incidence across all counties, separately by sex, are available in eTable 3 in Supplement 1 for female individuals and eTable 4 in Supplement 1 for male individuals.

## Discussion

Across 254 counties and 8 HSRs in Texas, HPV vaccination series initiation rates and up-to-date status as well as the incidence of HPV-related cancer varied widely. For both HPV-vaccine eligible female and male children and teenagers, the HPV vaccination series initiation and up-to-date status at the state, HSR, and county level has increased over time, which is consistent with findings of a study carried out in Florida.^14^ Some of the counties and HSRs in Texas are doing well in terms of coverage, whereas others are lagging. Overall, HSR 2/3 and HSR 4/5N and more counties in North Texas are far behind regarding HPV vaccination uptake compared with East, West, and South regions. These counties are important to target for preventive strategies, such as enhanced vaccine promotion, avoiding tobacco, regular screening, and early treatment of precancerous lesions because they had the highest incidence of HPV-related cancers with the lowest HPV vaccination initiation rates. HSR 9/10 and HSR 11 and more counties in South Texas appear to be performing better than their counterparts from East, West, and North regions, although still below the Healthy People 2030 goal. Findings of low HPV vaccination series initiation and up-to-date status with high HPV-related cancer burden in North Texas compared with East, West, and South regions raise concern, as they may indicate continued widening of existing health disparities between North Texas and other regions.

Findings of substantial county- and HSR-level variations in the proportion of individuals aged 9 to 17 years who initiated or were up to date for their HPV vaccination series in this study support previous similar intrastate studies conducted among individuals aged 13 to 17 years in the United States.^24,25^ The county-level disparities seen in the current study may reflect biodemographic, social, and geographic differences existing between the different regions of Texas.^13^ North Texas, for example, is known to have a larger proportion of non-Hispanic Black residents and larger average household sizes, with more of its residents dwelling in rural counties than the residents of South Texas.^26^ Prior studies^27,28^ have shown that disparities are present between rural and urban vaccination rates, with adolescents living in rural areas having the lowest HPV vaccination initiation and up-to-date rates. The vaccination policies in North Texas could also be a contributing factor to the low HPV vaccination initiation and up-to-date rates seen in the region compared with South Texas region.^29^ For instance, antivaccination advocacy groups such as Texans for Vaccine Choice are operating extensively to block legislation that limits vaccine exemptions.^30^ Additionally, in North Texas, antivaccination legislature is being proposed and promoted by local politicians,^31^ which has been shown to decrease the willingness of parents to have their children vaccinated against HPV.^32^ Moreover, the current study found that the average HPV vaccination initiation rate of Texas is significantly lower than the 62.6% national average for year 2022.^9^ This confirms findings of previous studies indicating that Texas has one of the lowest HPV vaccination rates in the United States.^10,13,27^

Generally, over the 16-year (2006-2022) study period for female individuals and 11-year (2011-2022) study period for male individuals, there was an upward trend in vaccination rate, except for a slight drop over the 3 years between 2014 and 2016 for some of the HSRs. The reason for the slight drop in vaccination could be because some children moved out of the counties and HSRs during the 3-year period stated above. Further investigation would be needed to gain full insight to the underlying causes of this change in coverage observed between 2014 and 2016.

Most of the counties with the highest HPV-related cancer rates in this study for female individuals also had the highest cancer rates for male individuals. Specifically, 29 counties had high HPV-related cancer IRs for both male (16.4-64.9 per 100 000) and female (23.8-154.2 per 100 000) individuals simultaneously. Moreover, geographic clustering was seen for HPV-related cancers within the state, as more of these counties are in the northern region. More research is needed to better understand factors associated with high burden of HPV-related cancers in these counties.

The county-level HPV vaccination initiation and up-to-date status and HPV-related cancer rates observed in this study indicate that despite increasing rates of HPV vaccination initiation and up-to-date status over the years in Texas, counties with a high burden of HPV-related cancers will benefit from enhanced prevention efforts, including but not limited to HPV vaccination series initiation and completion, cervical precancer screening, and early treatment of precancerous lesions. Linking increased HPV vaccination rates to decreased HPV-related cancer IRs for some counties will be hampered by the possibility of population relocation, given that there is a latency period of 10 to 20 years between contracting HR-HPV infection and cancer diagnosis.

### Limitations

This study has some important limitations to be considered when interpreting the findings. First, this study was limited by the data available because we used existing data, which was collected for a different purpose. We were not able to consider geographic units such as zip code and census tract^33^ that are smaller than county-level units for our analysis because we could not access HPV vaccination records at such levels. Also, we were unable to do separate calculations for more than one age group stratified by sex as well as take into consideration the HPV attributable fraction in our cancer IR calculations. Furthermore, no follow-up data, including but not limited to HR-HPV infection for individuals that were vaccinated against HPV, were available, and therefore, the causal link between HR-HPV infection and HPV-related cancers in our study population is difficult to establish. Second, regarding HPV-related cancer incidence, we removed some counties from our analysis due to overall low incidence of these cancers (<5 cases), making us unable to quantify and rank all counties with respect to HPV-related cancers. Third, only HPV vaccination data for consented clients with a valid Texas address were made available to us by Texas DSHS, which may result in our study’s sample not being a true representative of the population of individuals aged 9 to 17 years residing in Texas who were vaccinated against HPV during the study period. Fourth, the possibility of ecological fallacy cannot be excluded because our study was conducted at the area and county level and may not be attributable to individuals. Additionally, we acknowledge that county-level considerations may mask findings at the neighborhood level that would be apparent as smaller units; county-level assessment of HPV vaccination and HPV-related cancer IRs in the same analytic framework is novel.

## Conclusions

The incidence of HPV-related cancers varied widely across Texas counties and HSRs. The counties in the northern region had a higher IRs for HPV-related cancers and lower HPV vaccination rates compared with those in other regions. To address the disparities seen in the measures of interest in this study, designing and implementing targeted interventions to increase uptake and completion of the HPV vaccination series across counties and HSRs with low HPV vaccination initiation and up-to-date status are highly needed.

## Notes

Supplement 1. **eFigure.** Map Showing Health Service Regions of Texas **eTable 1.** Texas County-Level Estimates of HPV Metrics Among Female Individuals Aged 9 to 17 Years **eTable 2.** Texas County-Level Estimates of HPV Metrics Among Male Individuals Aged 9 to 17 Years **eTable 3.** Spearmen Correlation Matrix Between HPV Vaccination Initiation, HPV Vaccination Up-to-Date Status, and HPV-Related Cancer Incidence Across All Counties for Female Individuals **eTable 4.** Spearmen Correlation Matrix Between HPV Vaccination Initiation, HPV Vaccination Up-to-Date Status, and HPV-Related Cancer Incidence Across All Counties for Male Individuals

Supplement 2.
Data Sharing Statement
