# Supplementary material for: Human Papillomavirus Vaccination and Human Papillomavirus–Related Cancer Rates
Source: JAMA Netw Open. 2024 Sep 5;7(9):e2431807. doi: 10.1001/jamanetworkopen.2024.31807 (PMC11378004; doi:10.1001/jamanetworkopen.2024.31807)
Supplement: Supplement 1. — eFigure. Map Showing Health Service Regions of Texas eTable 1. Texas County-Level Estimates of HPV Metrics Among Female Individuals Aged 9 to 17 Years eTable 2. Texas County-Level Estimates of HPV Metrics Among Male Individuals Aged 9 to 17 Years eTable 3. Spearmen Correlation Matrix Between HPV Vaccination Initiation, HPV Vaccination Up-to-Date Status, and HPV-Related Cancer Incidence Across All Counties for Female Individuals eTable 4. Spearmen Correlation Matrix Between HPV Vaccination Initiation, HPV Vaccination Up-to-Date Status, and HPV-Related Cancer Incidence Across All Counties for Male Individuals [file jamanetwopen-e2431807-s001.pdf]

Supplemental Online Content

Adekanmbi V, Sokale I, Guo F, et al. Human papillomavirus vaccination and human papillomavirus–related cancer rates. *JAMA Netw Open.* 2024;7(9):e2431807.
doi:10.1001/jamanetworkopen.2024.31807

eFigure. Map Showing Health Service Regions of Texas

eTable 1. Texas County-Level Estimates of HPV Metrics Among Female Individuals Aged 9 to 17 Years

eTable 2. Texas County-Level Estimates of HPV Metrics Among Male Individuals Aged 9 to 17 Years

eTable 3. Spearmen Correlation Matrix Between HPV Vaccination Initiation, HPV Vaccination Up-to-Date Status, and HPV-Related Cancer Incidence Across All Counties for Female Individuals

eTable 4. Spearmen Correlation Matrix Between HPV Vaccination Initiation, HPV Vaccination Up-to-Date Status, and HPV-Related Cancer Incidence Across All Counties for Male Individuals

This supplemental material has been provided by the authors to give readers additional information about their work.

© 2024 Adekanmbi V et al. *JAMA Network Open.*

eFigure. Map Showing Health Service Regions of Texas

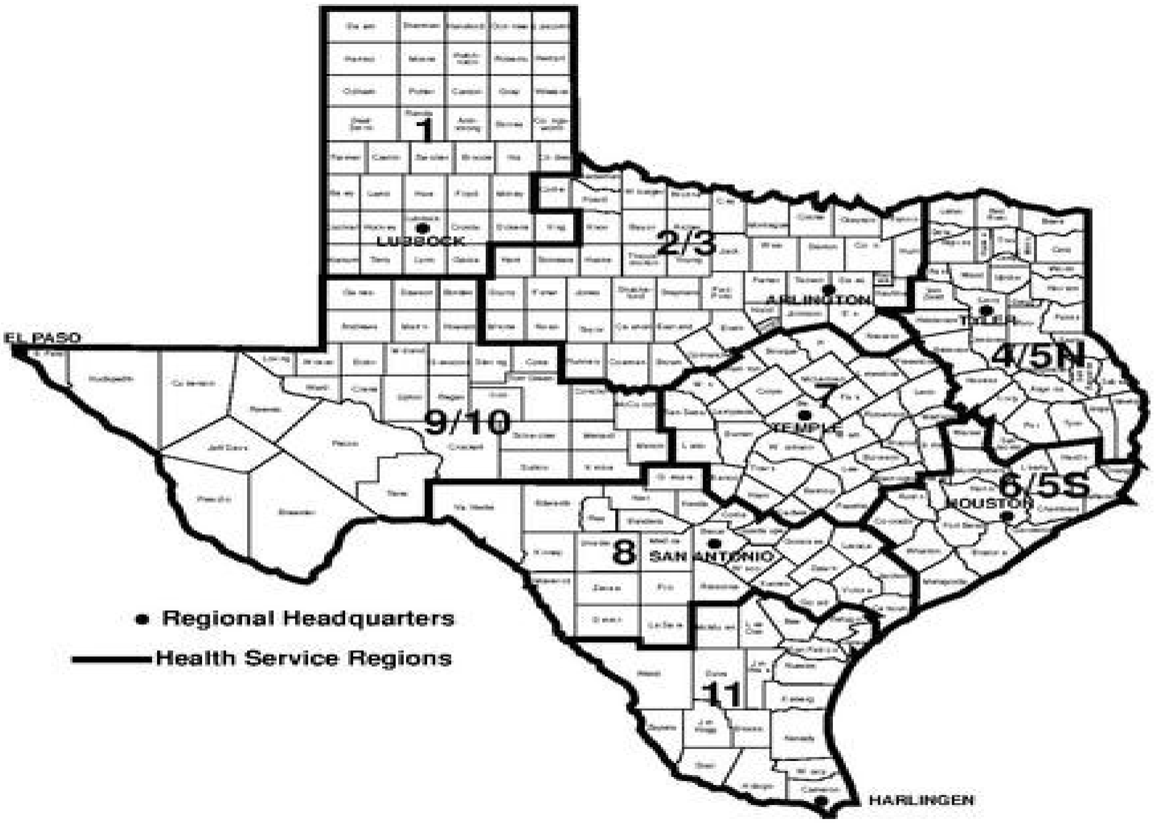

© 2024 Adekanmbi V et al. *JAMA Network Open.*

eTable 1. Texas County-Level Estimates of HPV Metrics Among Female Individuals Aged 9 to 17 Years

County	Proportion 9- to 17-year-olds who initiated the HPV vaccine 2021–2022[a]	Proportion of 9- to 17-year-olds who were up to date for the HPV vaccine 2021–2022[a]	HPV-related cancer incidence rate[b]
Anderson	35.2%	14.7%	18.6
Andrews	42.7%	24.5%	21.6
Angelina	44.4%	16.3%	26.1
Aransas	36.0%	14.0%	21.9
Archer	32.2%	10.5%	21.0
Armstrong	19.1%	8.8%	61.1
Atascosa	44.1%	18.8%	18.4
Austin	38.1%	17.3%	17.2
Bailey	30.6%	13.7%	0
Bandera	33.9%	16.4%	19.8
Bastrop	47.2%	19.7%	12.9
Baylor	46.1%	26.9%	70.0
Bee	51.6%	20.5%	33.0
Bell	29.0%	13.7%	23.7
Bexar	42.0%	18.5%	18.3
Blanco	30.7%	12.9%	22.7
Borden	41.0%	15.4%	154.6
Bosque	29.8%	15.8%	34.7
Bowie	24.4%	13.6%	22.2
Brazoria	44.0%	17.8%	17.6
Brazos	46.5%	19.6%	14.8
Brewster	30.0%	17.2%	33.4
Briscoe	59.1%	9.5%	19.9
Brooks	6.2%	1.6%	15.4
Brown	41.2%	19.4%	21.6
Burleson	46.3%	21.0%	19.2
Burnet	41.6%	17.6%	22.6
Caldwell	46.6%	20.9%	16.6
Calhoun	50.7%	17.2%	26.7
Callahan	40.0%	20.7%	10.5
Cameron	46.7%	15.7%	18.0
Camp	43.6%	19.2%	14.6
Carson	28.4%	13.2%	30.3
Cass	24.3%	11.5%	22.4
Castro	39.4%	12.9%	17.5
Chambers	41.7%	16.2%	9.1
Cherokee	39.2%	16.5%	23.1
Childress	31.9%	14.3%	46.7
Clay	31.8%	16.4%	16.8
Cochran	37.2%	18.8%	24.6
Coke	40.6%	19.7%	35.3
Coleman	44.0%	21.2%	14.4
Collin	23.6%	11.4%	11.7
Collingsworth	18.9%	8.9%	0
Colorado	31.7%	16.3%	35.3

© 2024 Adekanmbi V et al. *JAMA Network Open.*

Comal	34.2%	15.3%	19.4
Comanche	40.6%	16.7%	38.2
Concho	57.4%	26.6%	32.1
Cooke	28.2%	14.1%	20.2
Coryell	23.7%	11.0%	22.3
Cottle	47.4%	23.7%	50.0
Crane	35.0%	20.2%	11.4
Crockett	31.2%	14.6%	44.4
Crosby	44.7%	17.3%	26.4
Culberson	46.1%	28.1%	0
Dallam	25.4%	15.5%	22.1
Dallas	37.3%	18.5%	18.3
Dawson	20.6%	11.7%	8.5
DeWitt	47.2%	19.2%	25.5
Deaf Smith	42.0%	21.1%	10.8
Delta	33.0%	17.1%	28.9
Denton	29.2%	14.1%	14.4
Dickens	20.9%	10.5%	69.5
Dimmit	69.4%	30.4%	20.1
Donley	34.1%	16.6%	7.8
Duval	62.9%	22.7%	19.6
Eastland	26.1%	10.5%	25.2
Ector	34.8%	18.2%	26.7
Edwards	27.3%	11.6%	11.1
El Paso	47.7%	17.8%	17.6
Ellis	36.2%	16.7%	23.7
Erath	25.9%	12.1%	22.9
Falls	46.7%	20.8%	16.0
Fannin	36.0%	16.6%	21.3
Fayette	35.7%	14.2%	19.9
Fisher	55.6%	26.3%	52.3
Floyd	50.1%	23.8%	18.0
Foard	16.9%	10.8%	19.3
Fort Bend	43.4%	17.6%	12.8
Franklin	43.0%	17.2%	5.8
Freestone	30.2%	13.7%	29.2
Frio	11.7%	9.0%	25.3
Gaines	21.7%	11.3%	9.6
Galveston	45.8%	16.3%	20.5
Garza	39.4%	18.8%	0
Gillespie	26.4%	11.2%	19.0
Glasscock	43.4%	21.7%	27.9
Goliad	45.7%	16.5%	14.8
Gonzales	40.3%	21.9%	17.8
Gray	25.4%	12.4%	35.3
Grayson	34.2%	16.6%	22.3
Gregg	34.5%	15.3%	19.9
Grimes	45.3%	20.7%	16.8
Guadalupe	35.3%	15.0%	16.4
Hale	39.2%	19.9%	24.1

© 2024 Adekanmbi V et al. *JAMA Network Open.*

Hall	36.3%	15.4%	30.5
Hamilton	33.3%	17.8%	3.9
Hansford	26.9%	12.9%	29.6
Hardeman	38.4%	20.3%	0
Hardin	25.9%	14.1%	13.5
Harris	45.3%	18.0%	17.3
Harrison	39.7%	17.3%	23.2
Hartley	26.1%	16.7%	15.4
Haskell	38.8%	19.3%	58.8
Hays	42.2%	17.3%	18.4
Hemphill	28.1%	15.8%	16.9
Henderson	33.1%	15.4%	30.1
Hidalgo	40.4%	16.8%	20.2
Hill	33.0%	17.8%	34.7
Hockley	34.5%	17.5%	21.2
Hood	37.9%	15.9%	24.4
Hopkins	35.1%	14.4%	22.4
Houston	37.9%	15.2%	15.8
Howard	33.1%	18.0%	21.7
Hudspeth	50.5%	17.1%	28.5
Hunt	38.2%	16.6%	13.9
Hutchinson	23.1%	9.5%	30.8
Irion	35.5%	14.0%	43.3
Jack	32.5%	13.3%	30.3
Jackson	48.7%	20.0%	24.3
Jasper	23.7%	13.0%	26.6
Jeff Davis	34.0%	23.6%	0
Jefferson	35.2%	16.0%	19.7
Jim Hogg	56.5%	23.3%	9.0
Jim Wells	59.6%	20.4%	26.2
Johnson	32.3%	17.2%	21.6
Jones	49.9%	22.7%	28.3
Karnes	33.3%	19.3%	9.9
Kaufman	35.2%	16.7%	22.4
Kendall	27.4%	12.9%	14.9
Kenedy	53.3%	16.7%	0
Kent	30.4%	23.2%	45.4
Kerr	37.3%	17.4%	18.7
Kimble	38.3%	20.6%	10.6
King	13.8%	6.9%	0
Kinney	33.5%	18.0%	22.3
Kleberg	53.2%	16.9%	32.5
Knox	47.3%	26.1%	0
La Salle	36.3%	19.4%	6.5
Lamar	44.6%	20.0%	18.4
Lamb	37.4%	15.3%	24.8
Lampasas	28.1%	13.9%	40.4
Lavaca	46.7%	20.6%	13.2
Lee	48.6%	15.2%	20.2
Leon	37.9%	18.2%	26.6

© 2024 Adekanmbi V et al. *JAMA Network Open.*

Liberty	37.5%	16.5%	24.7
Limestone	38.2%	16.8%	22.0
Lipscomb	26.3%	10.7%	22.0
Live Oak	43.6%	20.4%	38.1
Llano	40.1%	18.0%	16.9
Loving	.	.	0
Lubbock	43.9%	17.5%	19.6
Lynn	38.8%	12.9%	25.9
Madison	44.8%	20.8%	17.1
Marion	34.4%	12.8%	36.4
Martin	27.9%	17.8%	15.4
Mason	39.8%	18.8%	18.5
Matagorda	50.5%	20.2%	17.9
Maverick	35.8%	14.1%	20.5
McCulloch	40.1%	16.8%	34.2
McLennan	46.6%	20.1%	19.5
McMullen	38.3%	25.5%	46.5
Medina	42.3%	20.2%	18.6
Menard	50.0%	16.7%	48.7
Midland	32.4%	16.1%	20.3
Milam	47.9%	19.5%	22.2
Mills	44.9%	16.0%	17.1
Mitchell	41.7%	23.8%	55.1
Montague	21.1%	9.8%	19.0
Montgomery	35.3%	15.0%	17.0
Moore	19.7%	10.5%	9.8
Morris	41.0%	14.7%	29.2
Motley	50.9%	23.6%	92.9
Nacogdoches	32.5%	13.8%	20.2
Navarro	31.8%	16.1%	19.7
Newton	20.3%	11.4%	30.8
Nolan	53.2%	23.9%	27.6
Nueces	52.6%	19.4%	15.5
Ochiltree	22.4%	10.9%	12.6
Oldham	33.3%	9.5%	19.1
Orange	26.9%	13.1%	21.8
Palo Pinto	28.4%	13.8%	21.1
Panola	18.1%	9.1%	21.0
Parker	32.4%	15.1%	22.1
Parmer	25.8%	11.2%	4.2
Pecos	51.8%	23.2%	30.7
Polk	39.7%	17.4%	35.8
Potter	36.3%	15.3%	23.8
Presidio	36.8%	21.1%	18.6
Rains	32.9%	13.1%	20.3
Randall	29.4%	11.7%	20.5
Reagan	33.6%	13.1%	0
Real	37.5%	12.9%	15.4
Red River	42.8%	18.4%	34.3
Reeves	47.9%	8.4%	26.6

© 2024 Adekanmbi V et al. *JAMA Network Open.*

Refugio	46.9%	23.6%	10.9
Roberts	21.1%	10.5%	128.3
Robertson	50.6%	22.4%	24.8
Rockwall	27.9%	13.1%	15.5
Runnels	49.4%	23.3%	33.4
Rusk	34.0%	14.8%	14.8
Sabine	20.0%	11.2%	21.1
San Augustine	28.0%	14.2%	3.0
San Jacinto	34.9%	18.0%	16.2
San Patricio	48.1%	17.5%	24.5
San Saba	44.0%	18.4%	21.1
Schleicher	29.0%	14.7%	23.6
Scurry	47.5%	26.2%	29.5
Shackelford	34.4%	17.9%	0
Shelby	21.7%	12.3%	11.7
Sherman	23.0%	11.1%	0
Smith	37.9%	17.7%	19.8
Somervell	29.1%	14.2%	17.2
Starr	31.0%	12.6%	19.6
Stephens	41.9%	17.8%	15.4
Sterling	40.1%	13.9%	33.1
Stonewall	45.7%	14.3%	0
Sutton	40.5%	17.7%	0
Swisher	42.4%	24.0%	12.6
Tarrant	36.1%	18.2%	18.4
Taylor	39.3%	17.4%	22.2
Terrell	28.6%	14.3%	0
Terry	36.9%	18.5%	16.3
Throckmorton	45.5%	22.2%	0
Titus	49.2%	18.4%	25.4
Tom Green	47.6%	16.8%	20.2
Travis	41.5%	17.2%	14.2
Trinity	43.4%	21.1%	25.2
Tyler	31.9%	15.8%	37.8
Upshur	33.4%	15.6%	20.2
Upton	42.6%	25.9%	0
Uvalde	43.6%	17.1%	16.9
Val Verde	30.3%	14.5%	14.2
Van Zandt	34.8%	15.6%	25.4
Victoria	52.0%	16.3%	23.9
Walker	32.3%	16.1%	28.8
Waller	38.6%	17.6%	31.6
Ward	21.5%	13.5%	34.6
Washington	46.9%	17.6%	14.6
Webb	39.0%	14.9%	16.2
Wharton	52.6%	20.1%	17.3
Wheeler	26.8%	13.4%	6.2
Wichita	30.9%	12.4%	32.8
Wilbarger	38.4%	19.2%	30.4
Willacy	50.3%	20.4%	20.1

© 2024 Adekanmbi V et al. *JAMA Network Open.*

Williamson	37.3%	16.0%	14.6
Wilson	32.7%	14.8%	21.5
Winkler	29.5%	18.5%	0
Wise	28.5%	13.6%	26.1
Wood	32.3%	15.0%	12.4
Yoakum	18.8%	10.9%	16.1.
Young	29.9%	13.1%	32.6
Zapata	47.8%	22.3%	9.0
Zavala	41.9%	26.7%	25.6

[a]Average percent of 2021 and 2022 Texas Immunization Registry count divided by census population for 9–17-year-olds, [b]2021–2022 annualized adjusted HPV cancer incidence rate per 100,000 population, adjusted by 2000 US population.
HPV, human papillomavirus.

© 2024 Adekanmbi V et al. *JAMA Network Open.*

eTable 2. Texas County-Level Estimates of HPV Metrics Among Male Individuals Aged 9 to 17 Years

County	Average percent of 9- to 17-year-olds who initiated the HPV vaccine 2021–2022[a]	Average percent of 9- to 17-year-olds who were up to date for the HPV vaccine 2021–2022[a]	HPV-related cancer incidence rate[b]
Anderson	28.2%	12.2%	12.8
Andrews	37.3%	19.9%	11.1
Angelina	41.7%	16.3%	13.6
Aransas	32.7%	13.8%	20.0
Archer	26.8%	12.5%	7.9
Armstrong	21.6%	5.0%	30.8
Atascosa	40.7%	17.5%	10.5
Austin	37.8%	17.3%	14.5
Bailey	33.5%	15.8%	13.0
Bandera	30.5%	13.5%	17.4
Bastrop	43.8%	17.7%	13.0
Baylor	44.3%	23.2%	27.8
Bee	47.3%	19.0%	20.6
Bell	27.6%	13.2%	14.9
Bexar	40.1%	17.6%	11.8
Blanco	28.0%	12.3%	14.3
Borden	25.4%	19.0%	50.9
Bosque	26.9%	13.3%	11.8
Bowie	22.5%	12.5%	18.3
Brazoria	41.5%	17.0%	13.7
Brazos	44.3%	19.4%	13.8
Brewster	23.0%	12.6%	5.9
Briscoe	47.4%	11.7%	0
Brooks	6.9%	2.1%	0
Brown	37.1%	18.9%	14.1
Burleson	44.3%	18.2%	9.8
Burnet	40.8%	17.4%	15.3
Caldwell	43.9%	18.8%	19.5
Calhoun	49.4%	18.0%	12.1
Callahan	38.2%	19.5%	96
Cameron	46.5%	14.4%	10.2
Camp	40.9%	16.5%	25.4
Carson	22.4%	13.1%	4.5
Cass	22.5%	11.3%	11.7
Castro	34.6%	14.7%	8.9
Chambers	39.7%	15.3%	18.1
Cherokee	38.3%	17.4%	18.6
Childress	26.6%	14.0%	5.8
Clay	28.9%	11.8%	21.3
Cochran	28.3%	11.0%	0
Coke	46.5%	19.1%	30.6
Coleman	37.4%	19.3%	13.5
Collin	21.6%	10.5%	12.2
Collingsworth	19.8%	12.5%	10.6
Colorado	32.1%	17.0%	18.7

© 2024 Adekanmbi V et al. *JAMA Network Open.*

Comal	32.6%	14.7%	17.2
Comanche	38.2%	16.8%	9.6
Concho	37.4%	14.6%	8.5
Cooke	25.8%	12.8%	8.7
Coryell	20.9%	10.2%	16.9
Cottle	56.5%	34.8%	45.9
Crane	39.9%	22.2%	0
Crockett	27.4%	16.4%	0
Crosby	38.8%	16.8%	10.7
Culberson	41.4%	22.2%	60.6
Dallam	21.1%	13.3%	12.9
Dallas	35.3%	18.0%	14.6
Dawson	16.9%	10.7%	17.0
DeWitt	43.2%	18.9%	9.4
Deaf Smith	34.4%	17.7%	10.0
Delta	32.6%	11.9%	35.7
Denton	27.4%	13.5%	12.1
Dickens	16.0%	11.8%	0
Dimmit	77.8%	24.6%	7.6
Donley	12.6%	8.0%	19.0
Duval	63.1%	21.8%	26.6
Eastland	25.6%	12.5%	9.1
Ector	31.9%	17.4%	10.9
Edwards	28.1%	14.4%	0
El Paso	46.2%	17.5%	8.7
Ellis	35.4%	16.5%	14.1
Erath	25.6%	12.1%	12.8
Falls	45.7%	23.5%	11.9
Fannin	31.6%	14.4%	10.0
Fayette	32.5%	13.5%	11.6
Fisher	54.5%	28.1%	31.2
Floyd	47.3%	22.3%	17.0
Foard	22.8%	14.0%	0
Fort Bend	41.6%	17.1%	10.6
Franklin	39.4%	13.9%	12.6
Freestone	30.2%	15.9%	13.3
Frio	11.6%	9.4%	7.1
Gaines	21.5%	10.9%	9.6
Galveston	43.0%	15.1%	20.8
Garza	34.6%	18.4%	0
Gillespie	22.2%	10.6%	11.0
Glasscock	39.7%	18.4%	0
Goliad	40.5%	15.0%	13.5
Gonzales	38.1%	19.4%	7.3
Gray	23.7%	12.5%	18.6
Grayson	31.8%	16.2%	17.4
Gregg	32.6%	14.4%	17.4
Grimes	44.4%	21.3%	19.4
Guadalupe	33.6%	14.8%	13.5
Hale	37.0%	21.3%	11.5

© 2024 Adekanmbi V et al. *JAMA Network Open.*

Hall	28.9%	11.4%	0
Hamilton	27.6%	15.6%	13.2
Hansford	27.2%	14.3%	20.2
Hardeman	48.0%	27.0%	60.1
Hardin	24.3%	13.6%	16.9
Harris	43.7%	17.6%	12.0
Harrison	35.0%	16.1%	19.5
Hartley	17.5%	11.5%	0
Haskell	49.9%	25.3%	11.0
Hays	40.4%	18.1%	13.8
Hemphill	22.6%	15.6%	0
Henderson	30.7%	14.7%	23.4
Hidalgo	38.2%	16.7%	8.0
Hill	29.5%	16.1%	27.9
Hockley	30.7%	15.4%	19.9
Hood	34.5%	13.6%	23.8
Hopkins	36.2%	17.7%	12.9
Houston	35.6%	15.0%	13.8
Howard	32.8%	17.5%	11.4
Hudspeth	52.9%	18.1%	18.6
Hunt	37.0%	16.4%	18.6
Hutchinson	17.2%	9.3%	10.6
Irion	35.7%	15.7%	0
Jack	29.2%	11.8%	19.6
Jackson	48.6%	19.8%	6.3
Jasper	19.6%	11.1%	11.0
Jeff Davis	25.8%	18.0%	0
Jefferson	30.9%	13.9%	14.2
Jim Hogg	57.6%	23.0%	30.4
Jim Wells	55.8%	20.4%	11.3
Johnson	29.0%	15.6%	13.1
Jones	44.4%	19.3%	11.6
Karnes	31.2%	21.2%	14.8
Kaufman	32.8%	16.1%	16.0
Kendall	25.1%	10.8%	12.0
Kenedy	48.4%	12.9%	0
Kent	25.0%	8.3%	38.6
Kerr	37.2%	18.1%	18.7
Kimble	36.5%	19.4%	10.9
King	33.3%	11.1%	0
Kinney	35.8%	15.5%	7.4
Kleberg	51.4%	17.8%	15.8
Knox	48.0%	21.6%	10.0
La Salle	31.3%	15.5%	6.0
Lamar	39.8%	18.0%	16.2
Lamb	39.2%	19.6%	17.5
Lampasas	25.2%	14.5%	13.6
Lavaca	43.3%	20.1%	15.6
Lee	49.4%	18.8%	9.7
Leon	32.6%	14.9%	9.7

© 2024 Adekanmbi V et al. *JAMA Network Open.*

Liberty	34.7%	16.1%	13.6
Limestone	38.8%	18.2%	18.0
Lipscomb	25.5%	7.6%	0
Live Oak	45.8%	21.7%	20.4
Llano	42.0%	18.3%	23.7
Loving	-	-	0
Lubbock	40.6%	16.6%	11.6
Lynn	41.0%	19.1%	22.8
Madison	43.4%	20.2%	7.3
Marion	30.4%	14.4%	27.3
Martin	28.7%	15.1%	45.4
Mason	28.0%	13.1%	5.3
Matagorda	48.3%	18.5%	17.1
Maverick	35.3%	15.0%	4.6
McCulloch	30.8%	14.7%	26.9
McLennan	43.5%	19.2%	17.8
McMullen	22.7%	4.5%	0
Medina	38.2%	19.3%	6.0
Menard	43.3%	17.2%	0
Midland	30.4%	16.3%	12.2
Milam	46.2%	18.4%	19.8
Mills	41.5%	17.8%	6.4
Mitchell	41.0%	22.2%	0
Montague	17.1%	8.4%	17.8
Montgomery	33.6%	14.4%	14.0
Moore	18.9%	9.2%	1.9
Morris	41.6%	16.7%	22.3
Motley	32.9%	30.0%	22.2
Nacogdoches	29.0%	12.3%	11.7
Navarro	29.6%	15.8%	19.6
Newton	16.1%	9.4%	11.7
Nolan	54.7%	25.7%	6.7
Nueces	50.8%	19.7%	13.9
Ochiltree	19.9%	10.7%	7.0
Oldham	29.7%	12.0%	0
Orange	23.1%	11.2%	21.6
Palo Pinto	24.0%	11.1%	29.0
Panola	15.0%	7.0%	14.1
Parker	30.1%	14.7%	17.1
Parmer	24.4%	11.5%	4.2
Pecos	44.3%	21.7%	3.1
Polk	37.4%	18.9%	23.1
Potter	32.0%	14.3%	19.8
Presidio	36.2%	20.5%	0
Rains	29.4%	12.7%	6.5
Randall	27.3%	11.7%	12.5
Reagan	33.1%	12.5%	22.5
Real	29.7%	16.4%	12.9
Red River	38.0%	18.7%	5.1
Reeves	48.9%	5.7%	14.6

© 2024 Adekanmbi V et al. *JAMA Network Open.*

Refugio	49.4%	21.6%	0
Roberts	22.5%	15.5%	0
Robertson	45.7%	19.0%	11.2
Rockwall	25.0%	11.6%	17.8
Runnels	46.8%	22.7%	35.1
Rusk	26.9%	13.5%	14.5
Sabine	14.6%	10.1%	30.3
San Augustine	24.4%	11.7%	18.3
San Jacinto	30.3%	14.2%	25.0
San Patricio	46.6%	17.7%	16.9
San Saba	40.2%	22.8%	8.7
Schleicher	30.6%	13.6%	13.9
Scurry	45.8%	26.3%	5.9
Shackelford	36.9%	15.4%	46.0
Shelby	18.0%	9.2%	7.7
Sherman	22.8%	9.0%	0
Smith	36.6%	18.2%	14.4
Somervell	28.5%	13.6%	16.3
Starr	29.1%	12.7%	5.9
Stephens	37.0%	18.7%	17.4
Sterling	35.2%	20.5%	62.5
Stonewall	38.0%	14.1%	64.9
Sutton	36.9%	18.4%	0
Swisher	40.0%	20.7%	9.5
Tarrant	34.3%	17.5%	15.1
Taylor	38.8%	17.2%	16.3
Terrell	15.3%	8.5%	35.5
Terry	35.4%	18.3%	90.0
Throckmorton	37.0%	14.0%	16.0
Titus	48.2%	18.0%	10.3
Tom Green	46.2%	16.9%	13.7
Travis	39.7%	17.2%	13.6
Trinity	42.4%	21.1%	24.2
Tyler	27.7%	14.4%	24.7
Upshur	28.4%	14.0%	20.7
Upton	42.8%	27.0%	0
Uvalde	39.5%	19.2%	14.2
Val Verde	27.0%	14.4%	7.1
Van Zandt	29.9%	13.8%	15.1
Victoria	51.4%	17.2%	16.5
Walker	29.8%	15.2%	22.6
Waller	38.7%	18.1%	16.6
Ward	21.0%	13.2%	6.8
Washington	42.5%	16.8%	15.0
Webb	37.4%	14.5%	7.5
Wharton	51.0%	21.2%	12.2
Wheeler	22.6%	13.5%	13.7
Wichita	27.6%	11.3%	13.9
Wilbarger	33.6%	18.6%	3.0
Willacy	53.1%	34.9%	5.1

© 2024 Adekanmbi V et al. *JAMA Network Open.*

Williamson	35.4%	15.5%	13.4
Wilson	30.9%	15.2%	7.8
Winkler	25.4%	13.3%	4.6
Wise	25.5%	11.7%	11.1
Wood	30.4%	14.7%	16.8
Yoakum	12.6%	6.8%	17.2
Young	25.0%	15.0%	28.7
Zapata	45.9%	21.6%	7.2
Zavala	42.7%	28.4%	12.7

[a]Average percent of 2021 and 2022 Texas Immunization Registry count divided by census population for 9–17-year-olds, [b]2021–2022 annualized adjusted HPV cancer incidence rate per 100,000 population, adjusted by 2000 US population.
HPV, human papillomavirus.

© 2024 Adekanmbi V et al. *JAMA Network Open.*

eTable 3. Spearmen Correlation Matrix Between HPV Vaccination Initiation, HPV Vaccination Up-to-Date Status, and HPV-Related Cancer Incidence Across All Counties for Female Individuals

County	pctini	pctutd	rate	pctini_rank	pctutd_rank	rate_rank
Anderson	35.2%	14.7%	18.6	146	175	151
Andrews	42.7%	24.5%	21.6	75	11	110
Angelina	44.4%	16.3%	26.1	61	140	64
Aransas	36.0%	14.0%	21.9	137	190	105
Archer	32.2%	10.5%	21.0	181	240	118
Armstrong	19.1%	8.8%	61.1	246	250	5
Atascosa	44.1%	18.8%	18.4	62	68	155
Austin	38.1%	17.3%	17.2	118	109	169
Bailey	30.6%	13.7%	0.0	191	196	245
Bandera	33.9%	16.4%	19.8	161	137	135
Bastrop	47.2%	19.7%	12.9	39	56.5	208
Baylor	46.1%	26.9%	6.9	49	3	229
Bee	51.6%	20.5%	33.0	15	43	33
Bell	29.0%	13.7%	23.7	203	195	84
Bexar	42.0%	18.5%	18.3	80	72	159
Blanco	30.7%	12.9%	22.7	190	212	90
Borden	41.0%	15.4%	154.2	89	158.5	1
Bosque	29.8%	15.8%	34.7	197	150	25
Bowie	24.4%	13.6%	22.3	228	198	98
Brazoria	44.0%	17.8%	17.6	63	90	164
Brazos	46.5%	19.6%	14.8	47	58	195
Brewster	30.0%	17.2%	33.4	195	112	31
Briscoe	59.1%	9.5%	19.9	4	246	133
Brooks	6.2%	1.6%	15.4	253	253	192
Brown	41.2%	19.4%	21.7	88	61	108
Burleson	46.3%	21.0%	19.2	48	35	145
Burnet	41.6%	17.6%	22.6	86	99	91
Caldwell	46.6%	20.9%	16.6	46	36	179
Calhoun	50.7%	17.2%	26.7	17	110	58
Callahan	40.0%	20.7%	10.5	99	39	220
Cameron	46.7%	15.7%	18.0	44	153	161
Camp	43.6%	19.2%	14.5	68	67	199
Carson	28.4%	13.2%	30.3	206	203	45
Cass	24.3%	11.5%	22.3	229	225	94
Castro	39.4%	12.9%	17.5	103	213	166
Chambers	41.7%	16.2%	9.1	84	142	224
Cherokee	39.2%	16.5%	23.1	107	133	88
Childress	31.9%	14.3%	46.7	183	181	11
Clay	31.8%	16.4%	16.8	184	136	177
Cochran	37.2%	18.8%	24.6	129	70	77

© 2024 Adekanmbi V et al. *JAMA Network Open.*

Coke	40.6%	19.7%	35.3	91	56.5	23
Coleman	44.0%	21.2%	14.4	64	31	201
Collin	23.6%	11.4%	11.6	232	226	214
Collingsworth	18.9%	8.9%	0.0	247	249	245
Colorado	31.7%	16.3%	35.3	186	138	22
Comal	34.2%	15.3%	19.4	157	162	143
Comanche	40.6%	16.7%	38.2	92	125	17
Concho	57.4%	26.6%	32.1	5	5	37
Cooke	28.2%	14.1%	20.2	208	188	130
Coryell	23.7%	11.0%	22.3	230	233	95
Cottle	47.4%	23.7%	50.0	36	16	9
Crane	35.0%	20.2%	11.4	148	48	215
Crockett	31.2%	14.6%	44.4	187	176	14
Crosby	44.7%	17.3%	26.4	59	108	62
Culberson	46.1%	28.1%	0.0	50	2	245
Dallam	25.4%	15.5%	22.1	227	156	102
Dallas	37.3%	18.5%	18.3	127	74	158
Dawson	20.6%	11.7%	8.5	242	222	227
DeWitt	47.2%	19.2%	25.5	38	66	68
Deaf Smith	42.0%	21.1%	10.8	81	34	218
Delta	33.0%	17.1%	28.9	170	116	51
Denton	29.2%	14.1%	14.4	200	187	202
Dickens	20.9%	10.5%	69.5	241	241	4
Dimmit	69.4%	30.4%	20.1	1	1	131
Donley	34.1%	16.6%	7.8	158	130	228
Duval	62.9%	22.7%	19.6	2	24	141
Eastland	26.1%	10.5%	25.2	222	239	73
Ector	34.8%	18.2%	26.7	150	79	57
Edwards	27.3%	11.6%	11.1	215	224	216
El Paso	47.7%	17.8%	17.6	33	93	165
Ellis	36.2%	16.7%	23.7	135	123.5	85
Erath	25.9%	12.1%	22.9	223	221	89
Falls	46.7%	20.8%	16.0	42	38	185
Fannin	36.0%	16.6%	21.3	138	129	112
Fayette	35.7%	14.2%	19.9	140	184	132
Fisher	55.6%	26.3%	52.3	7	6	8
Floyd	50.1%	23.8%	18.0	22	14	160
Foard	16.9%	10.8%	19.3	250	236	144
Fort Bend	43.4%	17.6%	12.8	70	96	209
Franklin	43.0%	17.2%	5.8	73	114	232
Freestone	30.2%	13.7%	29.2	194	197	49
Frio	11.7%	9.0%	25.3	252	248	71
Gaines	21.7%	11.3%	9.6	237	228	223

© 2024 Adekanmbi V et al. *JAMA Network Open.*

Galveston	45.8%	16.3%	20.5	51	141	121
Garza	39.4%	18.8%	0.0	104	69	245
Gillespie	26.4%	11.2%	19.0	219	231	148
Glasscock	43.4%	21.7%	27.9	72	30	55
Goliad	45.7%	16.5%	14.8	53	135	196
Gonzales	40.3%	21.9%	17.8	95	29	163
Gray	25.4%	12.4%	35.3	226	219	24
Grayson	34.2%	16.6%	22.3	156	131	97
Gregg	34.5%	15.3%	19.9	153	161	134
Grimes	45.3%	20.7%	16.8	56	40	178
Guadalupe	35.3%	15.0%	16.4	143	167	180
Hale	39.2%	19.9%	24.1	106	55	81
Hall	36.3%	15.4%	30.5	134	158.5	42
Hamilton	33.3%	17.8%	3.9	167	91	234
Hansford	26.9%	12.9%	29.6	216	210	47
Hardeman	38.4%	20.3%	0.0	112	47	245
Hardin	25.9%	14.1%	13.5	224	185	206
Harris	45.3%	18.0%	17.3	55	85	168
Harrison	39.7%	17.3%	23.2	101	107	87
Hartley	26.1%	16.7%	15.4	221	123.5	189
Haskell	38.8%	19.3%	58.8	110	63	6
Hays	42.2%	17.3%	18.3	79	106	157
Hemphill	28.1%	15.8%	16.9	210	151	176
Henderson	33.1%	15.4%	30.1	168	157	46
Hidalgo	40.4%	16.8%	20.2	94	119	125
Hill	33.0%	17.8%	34.7	171	89	26
Hockley	34.5%	17.5%	21.2	152	102	113
Hood	37.9%	15.9%	24.4	122	149	79
Hopkins	35.1%	14.4%	22.4	147	178	93
Houston	37.9%	15.2%	15.8	119.5	165	186
Howard	33.1%	18.0%	21.7	169	86	107
Hudspeth	50.5%	17.1%	28.5	20	117	53
Hunt	38.2%	16.6%	13.9	117	132	205
Hutchinson	23.1%	9.5%	30.8	233	245	39
Irion	35.5%	14.0%	43.3	141	189	15
Jack	32.5%	13.3%	30.3	175	202	44
Jackson	48.7%	20.0%	24.3	27	53	80
Jasper	23.7%	13.0%	26.6	231	209	60
Jeff Davis	34.0%	23.6%	0.0	160	19	245
Jefferson	35.2%	16.0%	19.7	145	148	137
Jim Hogg	56.5%	23.3%	9.0	6	21	225
Jim Wells	59.6%	20.4%	26.2	3	45	63

© 2024 Adekanmbi V et al. *JAMA Network Open.*

Johnson	32.3%	17.2%	21.6	179	111	109
Jones	49.9%	22.7%	28.3	24	25	54
Karnes	33.3%	19.3%	9.9	166	64	221
Kaufman	35.2%	16.7%	22.4	144	128	92
Kendall	27.4%	12.9%	14.9	214	211	194
Kenedy	53.3%	16.7%	0.0	8	126.5	245
Kent	30.4%	23.2%	45.4	192	22	13
Kerr	37.3%	17.4%	18.7	128	104	149
Kimble	38.3%	20.6%	10.6	115	42	219
King	13.8%	6.9%	0.0	251	252	245
Kinney	33.5%	18.0%	22.3	163	84	96
Kleberg	53.2%	16.9%	32.5	10	118	36
Knox	47.3%	26.1%	0.0	37	8	245
La Salle	36.3%	19.4%	6.5	133	62	230
Lamar	44.6%	20.0%	18.4	60	54	156
Lamb	37.4%	15.3%	24.8	125	163	75
Lampasas	28.1%	13.9%	40.4	209	191.5	16
Lavaca	46.7%	20.6%	13.2	43	41	207
Lee	48.6%	15.2%	20.2	28	164	127
Leon	37.9%	18.2%	26.6	121	80	61
Liberty	37.5%	16.5%	24.6	124	134	76
Limestone	38.2%	16.8%	22.0	116	120	104
Lipscomb	26.3%	10.7%	22.0	220	237	103
Live Oak	43.6%	20.4%	38.1	67	44	18
Llano	40.1%	18.0%	16.9	96	83	174
Loving	.	.	0.0			245
Lubbock	43.9%	17.5%	19.6	66	100	140
Lynn	38.8%	12.9%	25.9	109	214	65
Madison	44.8%	20.8%	17.1	58	37	171
Marion	34.4%	12.8%	36.4	154	216	20
Martin	27.9%	17.8%	15.3	213	92	193
Mason	39.8%	18.8%	18.5	100	71	153
Matagorda	50.5%	20.2%	17.9	19	49	162
Maverick	35.8%	14.1%	20.5	139	186	122
McCulloch	40.1%	16.8%	34.2	98	122	29
McLennan	46.6%	20.1%	19.5	45	51	142
McMullen	38.3%	25.5%	46.5	114	10	12
Medina	42.3%	20.2%	18.6	78	50	150
Menard	50.0%	16.7%	48.7	23	126.5	10
Midland	32.4%	16.1%	20.3	176	144	124
Milam	47.9%	19.5%	22.2	30	59	99
Mills	44.9%	16.0%	17.1	57	146	172

© 2024 Adekanmbi V et al. *JAMA Network Open.*

Mitchell	41.7%	23.8%	55.1	85	15	7
Montague	21.1%	9.8%	19.0	239	243	147
Montgomery	35.3%	15.0%	17.0	142	169	173
Moore	19.7%	10.5%	9.8	245	242	222
Morris	41.0%	14.7%	29.2	90	174	50
Motley	50.9%	23.6%	92.9	16	17	3
Nacogdoches	32.5%	13.8%	20.2	174	194	129
Navarro	31.8%	16.1%	19.7	185	145	139
Newton	20.3%	11.4%	30.8	243	227	40
Nolan	53.2%	23.9%	27.6	9	13	56
Nueces	52.6%	19.4%	15.5	12	60	188
Ochiltree	22.4%	10.9%	12.6	235	235	210
Oldham	33.3%	9.5%	19.1	165	244	146
Orange	26.9%	13.1%	21.8	217	205	106
Palo Pinto	28.4%	13.8%	21.1	207	193	116
Panola	18.1%	9.1%	21.0	249	247	117
Parker	32.4%	15.1%	22.1	177	166	101
Parmer	25.8%	11.2%	4.2	225	229.5	233
Pecos	51.8%	23.2%	30.7	14	23	41
Polk	39.7%	17.4%	35.8	102	103	21
Potter	36.3%	15.3%	23.8	132	160	83
Presidio	36.8%	21.1%	18.6	131	33	152
Rains	32.9%	13.1%	20.3	172	204	123
Randall	29.4%	11.7%	20.5	199	223	120
Reagan	33.6%	13.1%	0.0	162	206	245
Real	37.5%	12.9%	15.4	123	215	190
Red River	42.8%	18.4%	34.3	74	77	28
Reeves	47.9%	8.4%	26.6	31	251	59
Refugio	46.9%	23.6%	10.9	40	18	217
Roberts	21.1%	10.5%	128.3	240	238	2
Robertson	50.6%	22.4%	24.8	18	26	74
Rockwall	27.9%	13.1%	15.5	212	207	187
Runnels	49.4%	23.3%	33.4	25	20	30
Rusk	34.0%	14.8%	14.8	159	172	197
Sabine	20.0%	11.2%	21.1	244	229.5	115
San Augustine	28.0%	14.2%	3.0	211	182	235
San Jacinto	34.9%	18.0%	16.2	149	82	183
San Patricio	48.1%	17.5%	24.5	29	101	78
San Saba	44.0%	18.4%	21.1	65	76	114
Schleicher	29.0%	14.7%	23.6	202	173	86
Scurry	47.5%	26.2%	29.4	35	7	48
Shackelford	34.4%	17.9%	0.0	155	87	245

© 2024 Adekanmbi V et al. *JAMA Network Open.*

Shelby	21.7%	12.3%	11.7	236	220	213
Sherman	23.0%	11.1%	0.0	234	232	245
Smith	37.9%	17.7%	19.8	119.5	95	136
Somervell	29.1%	14.2%	17.2	201	183	170
Starr	31.0%	12.6%	19.7	188	217	138
Stephens	41.9%	17.8%	15.4	82	88	191
Sterling	40.1%	13.9%	33.1	97	191.5	32
Stonewall	45.7%	14.3%	0.0	52	179.5	245
Sutton	40.5%	17.7%	0.0	93	94	245
Swisher	42.4%	24.0%	12.6	77	12	211
Tarrant	36.1%	18.2%	18.4	136	81	154
Taylor	39.3%	17.4%	22.2	105	105	100
Terrell	28.6%	14.3%	0.0	204	179.5	245
Terry	36.9%	18.5%	16.3	130	75	181
Throckmorton	45.5%	22.2%	0.0	54	28	245
Titus	49.2%	18.4%	25.4	26	78	69
Tom Green	47.6%	16.8%	20.2	34	121	128
Travis	41.5%	17.2%	14.2	87	113	203
Trinity	43.4%	21.1%	25.2	71	32	72
Tyler	31.9%	15.8%	37.8	182	152	19
Upshur	33.4%	15.6%	20.2	164	155	126
Upton	42.6%	25.9%	0.0	76	9	245
Uvalde	43.6%	17.1%	16.9	69	115	175
Val Verde	30.3%	14.5%	14.2	193	177	204
Van Zandt	34.8%	15.6%	25.4	151	154	70
Victoria	52.0%	16.3%	23.9	13	139	82
Walker	32.3%	16.1%	28.8	180	143	52
Waller	38.6%	17.6%	31.6	111	97	38
Ward	21.5%	13.5%	34.6	238	200	27
Washington	46.9%	17.6%	14.5	41	98	198
Webb	39.0%	14.9%	16.2	108	170	182
Wharton	52.6%	20.1%	17.3	11	52	167
Wheeler	26.8%	13.4%	6.2	218	201	231
Wichita	30.9%	12.4%	32.7	189	218	34
Wilbarger	38.4%	19.2%	30.4	113	65	43
Willacy	50.3%	20.4%	20.5	21	46	119
Williamson	37.3%	16.0%	14.5	126	147	200
Wilson	32.7%	14.8%	21.5	173	171	111
Winkler	29.5%	18.5%	0.0	198	73	245
Wise	28.5%	13.6%	25.6	205	199	66
Wood	32.3%	15.0%	12.4	178	168	212
Yoakum	18.8%	10.9%	16.1	248	234	184
Young	29.9%	13.1%	32.6	196	208	35

© 2024 Adekanmbi V et al. *JAMA Network Open.*

Zapata	47.8%	22.3%	9.0	32	27	226
Zavala	41.9%	26.7%	25.6	83	4	67

Spearman correlation coefficient **0.02** **-0.05**

P value **0.73** **0.45**

Pctini: percentage of clients with at least one dose of HPV vaccine given the demographic grouping.

Pctutd: Percentage of clients that are up to date on their HPV series given their demographic grouping and age of initiation.

Rate: HPV-related cancer incidence rate.

pctini_rank: rank of % initiation.

pctutd_rank: rank of % up-to-date.

rate_rank: rank of cancer incidence rate.

© 2024 Adekanmbi V et al. *JAMA Network Open.*

eTable 4. Spearmen Correlation Matrix Between HPV Vaccination Initiation, HPV Vaccination Up-to-Date Status, and HPV-Related Cancer Incidence Across All Counties for Male Individuals

County	pctini	pctutd	rate	pctini_rank	pctutd_rank	rate_rank
Anderson	28.2%	12.2%	12.8	183	207	135
Andrews	37.3%	19.9%	11.1	102	38	160
Angelina	41.7%	16.3%	13.5	60	118	122
Aransas	32.7%	13.8%	20.0	140	177	45
Archer	26.8%	12.5%	7.9	198	204	193
Armstrong	21.6%	5.0%	30.8	231	251	14
Atascosa	40.7%	17.5%	10.0	70	94	173
Austin	37.8%	17.3%	14.5	97	98	101
Bailey	33.5%	15.8%	13.0	134	126	130
Bandera	30.5%	13.5%	17.4	159	184	71
Bastrop	43.8%	17.7%	13.0	47	87	131
Baylor	44.3%	23.2%	27.8	42	13	21
Bee	47.3%	19.0%	20.6	26	54	42
Bell	27.6%	13.2%	14.8	188	190	96
Bexar	40.1%	17.6%	11.8	75	89	149
Blanco	28.0%	12.3%	14.3	186	206	103
Borden	25.4%	19.0%	50.9	206	53	5
Bosque	26.9%	13.3%	11.8	197	189	148
Bowie	22.5%	12.5%	18.3	226	200	61
Brazoria	41.5%	17.0%	13.7	64	105	117
Brazos	44.3%	19.4%	13.8	43	43	114
Brewster	23.0%	12.6%	5.9	219	198	212.5
Briscoe	47.4%	11.7%	0.0	25	217	240
Brooks	6.9%	2.1%	0.0	253	253	240
Brown	37.1%	18.9%	14.1	104	57	108
Burleson	44.3%	18.2%	9.8	44	73	177
Burnet	40.8%	17.4%	15.3	69	97	92
Caldwell	43.9%	18.8%	19.5	46	59	51
Calhoun	49.4%	18.0%	12.1	15	80	144
Callahan	38.2%	19.5%	9.6	91	42	181
Cameron	46.5%	14.4%	10.2	31	158	172
Camp	40.9%	16.5%	25.4	68	114	25
Carson	22.4%	13.1%	4.5	228	193	221
Cass	22.5%	11.3%	11.7	227	222	151
Castro	34.6%	14.7%	8.9	127	153	187
Chambers	39.7%	15.3%	18.1	81	138	63
Cherokee	38.3%	17.4%	18.6	89	96	59
Childress	26.6%	14.0%	5.8	199	173	215
Clay	28.9%	11.8%	21.3	178	212	39

© 2024 Adekanmbi V et al. *JAMA Network Open.*

Cochran	28.3%	11.0%	0.0	182	228	240
Coke	46.5%	19.1%	30.6	30	51	15
Coleman	37.4%	19.3%	13.3	100	46	126
Collin	21.6%	10.5%	12.2	230	234	140
Collingsworth	19.8%	12.5%	10.6	237	202	170
Colorado	32.1%	17.0%	18.7	146	104	55
Comal	32.6%	14.7%	17.2	143	154	73
Comanche	38.2%	16.8%	9.6	92	107	182
Concho	37.4%	14.6%	8.5	99	155	191
Cooke	25.8%	12.8%	8.7	201	195	188
Coryell	20.9%	10.2%	16.9	235	235	81
Cottle	56.5%	34.8%	45.9	4	2	7
Crane	39.9%	22.2%	0.0	77	20	240
Crockett	27.4%	16.4%	0.0	191	115	240
Crosby	38.8%	16.8%	10.7	86	108	167
Culberson	41.4%	22.2%	60.6	65	18.5	3
Dallam	21.1%	13.3%	12.9	233	187.5	133
Dallas	35.3%	18.0%	14.6	122	78	98
Dawson	16.9%	10.7%	17.0	244	231	78
DeWitt	43.2%	18.9%	9.4	53	58	184
Deaf Smith	34.4%	17.7%	10.0	129	88	176
Delta	32.6%	11.9%	35.6	141	210	10
Denton	27.4%	13.5%	12.1	192	182	143
Dickens	16.0%	11.8%	0.0	246	211	240
Dimmit	77.8%	24.6%	7.6	1	11	196
Donley	12.6%	8.0%	19.0	250	246	54
Duval	63.1%	21.8%	26.6	2	21	24
Eastland	25.6%	12.5%	9.1	202	203	185
Ector	31.9%	17.4%	10.9	148	95	166
Edwards	28.1%	14.4%	0.0	184	161	240
El Paso	46.2%	17.5%	8.7	33	92	189
Ellis	35.4%	16.5%	14.1	120	113	107
Erath	25.6%	12.1%	12.8	203	208	136
Falls	45.7%	23.5%	11.9	38	12	147
Fannin	31.6%	14.4%	10.0	150	160	175
Fayette	32.5%	13.5%	11.5	145	185	155
Fisher	54.5%	28.1%	31.2	7	5	13
Floyd	47.3%	22.3%	17.0	27	17	77
Foard	22.8%	14.0%	0.0	221	170	240
Fort Bend	41.6%	17.1%	10.6	62	103	169
Franklin	39.4%	13.9%	12.6	83	175	138
Freestone	30.2%	15.9%	13.3	164	125	127

© 2024 Adekanmbi V et al. *JAMA Network Open.*

Frio	11.6%	9.4%	7.1	252	237	203
Gaines	21.5%	10.9%	9.6	232	229	180
Galveston	43.0%	15.1%	20.8	54	141	40
Garza	34.6%	18.4%	0.0	126	67	240
Gillespie	22.2%	10.6%	10.9	229	233	164
Glasscock	39.7%	18.4%	0.0	80	68	240
Goliad	40.5%	15.0%	13.5	72	146	123
Gonzales	38.1%	19.4%	7.3	94	44	200
Gray	23.7%	12.5%	18.6	217	199	57
Grayson	31.8%	16.2%	17.4	149	120	70
Gregg	32.6%	14.4%	17.4	144	159	69
Grimes	44.4%	21.3%	19.3	40	27	53
Guadalupe	33.6%	14.8%	13.5	133	148	124
Hale	37.0%	21.3%	11.5	107	28	156
Hall	28.9%	11.4%	0.0	177	221	240
Hamilton	27.6%	15.6%	13.2	189	131	128
Hansford	27.2%	14.3%	20.2	194	167	44
Hardeman	48.0%	27.0%	60.1	24	7	4
Hardin	24.3%	13.6%	16.9	215	178	80
Harris	43.7%	17.6%	12.0	48	90	145
Harrison	35.0%	16.1%	19.5	124	121	52
Hartley	17.5%	11.5%	0.0	241	220	240
Haskell	49.9%	25.3%	11.0	14	10	163
Hays	40.4%	18.1%	13.8	73	75	115
Hemphill	22.6%	15.6%	0.0	223	130	240
Henderson	30.7%	14.7%	23.4	157	152	31
Hidalgo	38.2%	16.7%	8.0	93	111	192
Hill	29.5%	16.1%	27.9	171	124	20
Hockley	30.7%	15.4%	19.9	156	137	46
Hood	34.5%	13.6%	23.8	128	180	29
Hopkins	36.2%	17.7%	12.9	114	86	134
Houston	35.6%	15.0%	13.8	117	145	113
Howard	32.8%	17.5%	11.4	138	93	157
Hudspeth	52.9%	18.1%	18.6	9	74	58
Hunt	37.0%	16.4%	18.6	108	116	60
Hutchinson	17.2%	9.3%	10.6	242	239	168
Irion	35.7%	15.7%	0.0	116	128	240
Jack	29.2%	11.8%	19.6	173	213	49
Jackson	48.6%	19.8%	6.3	19	39	209
Jasper	19.6%	11.1%	11.0	238	225	162
Jeff Davis	25.8%	18.0%	0.0	200	81	240
Jefferson	30.9%	13.9%	14.2	153	174	105

© 2024 Adekanmbi V et al. *JAMA Network Open.*

Jim Hogg	57.6%	23.0%	30.4	3	14	16
Jim Wells	55.8%	20.4%	11.3	5	35	158
Johnson	29.0%	15.6%	13.1	176	129	129
Jones	44.4%	19.3%	11.6	41	47	153
Karnes	31.2%	21.2%	14.8	152	29	97
Kaufman	32.8%	16.1%	16.0	139	123	89
Kendall	25.1%	10.8%	12.0	209	230	146
Kenedy	48.4%	12.9%	0.0	20	194	240
Kent	25.0%	8.3%	38.6	212	245	9
Kerr	37.2%	18.1%	18.7	103	77	56
Kimble	36.5%	19.4%	10.9	112	45	165
King	33.3%	11.1%	0.0	135	226	240
Kinney	35.8%	15.5%	7.4	115	132	198
Kleberg	51.4%	17.8%	15.8	11	84	90
Knox	48.0%	21.6%	10.0	23	24	174
La Salle	31.3%	15.5%	6.0	151	135	211
Lamar	39.8%	18.0%	16.2	78	82	87
Lamb	39.2%	19.6%	17.5	84	41	68
Lampasas	25.2%	14.5%	13.6	208	157	120
Lavaca	43.3%	20.1%	15.6	52	37	91
Lee	49.4%	18.8%	9.7	16	60	179
Leon	32.6%	14.9%	9.7	142	147	178
Liberty	34.7%	16.1%	13.6	125	122	121
Limestone	38.8%	18.2%	18.0	87	72	64
Lipscomb	25.5%	7.6%	0.0	205	247	240
Live Oak	45.8%	21.7%	20.4	37	22	43
Llano	42.0%	18.3%	23.7	59	69	30
Loving	.	.	0.0			240
Lubbock	40.6%	16.6%	11.6	71	112	154
Lynn	41.0%	19.1%	22.8	66	52	33
Madison	43.4%	20.2%	7.3	50	36	199
Marion	30.4%	14.4%	27.3	162	164	22
Martin	28.7%	15.1%	45.4	179	142	8
Mason	28.0%	13.1%	5.3	185	192	216
Matagorda	48.3%	18.5%	17.1	21	64	75
Maverick	35.3%	15.0%	4.6	121	143	219
McCulloch	30.8%	14.7%	26.9	155	150	23
McLennan	43.5%	19.2%	17.7	49	50	67
McMullen	22.7%	4.5%	0.0	222	252	240
Medina	38.2%	19.3%	6.0	90	48	210
Menard	43.3%	17.2%	0.0	51	100	240
Midland	30.4%	16.3%	12.2	160	119	142
Milam	46.2%	18.4%	19.7	34	65	48

© 2024 Adekanmbi V et al. *JAMA Network Open.*

Mills	41.5%	17.8%	6.3	63	83	208
Mitchell	41.0%	22.2%	0.0	67	18.5	240
Montague	17.1%	8.4%	17.8	243	244	65
Montgomery	33.6%	14.4%	14.0	132	162	109
Moore	18.9%	9.2%	1.9	239	241	225
Morris	41.6%	16.7%	22.3	61	110	36
Motley	32.9%	30.0%	22.2	137	3	37
Nacogdoches	29.0%	12.3%	11.7	175	205	152
Navarro	29.6%	15.8%	19.6	170	127	50
Newton	16.1%	9.4%	11.7	245	238	150
Nolan	54.7%	25.7%	6.7	6	9	206
Nueces	50.8%	19.7%	13.9	13	40	112
Ochiltree	19.9%	10.7%	7.0	236	232	204
Oldham	29.7%	12.0%	0.0	169	209	240
Orange	23.1%	11.2%	21.6	218	224	38
Palo Pinto	24.0%	11.1%	29.0	216	227	18
Panola	15.0%	7.0%	14.1	248	248	106
Parker	30.1%	14.7%	17.1	165	151	76
Parmer	24.4%	11.5%	4.2	213	219	222
Pecos	44.3%	21.7%	3.1	45	23	223
Polk	37.4%	18.9%	23.1	98	56	32
Potter	32.0%	14.3%	19.8	147	166	47
Presidio	36.2%	20.5%	0.0	113	34	240
Rains	29.4%	12.7%	6.5	172	197	207
Randall	27.3%	11.7%	12.5	193	216	139
Reagan	33.1%	12.5%	22.5	136	201	35
Real	29.7%	16.4%	12.9	168	117	132
Red River	38.0%	18.7%	5.1	96	61	218
Reeves	48.9%	5.7%	14.6	18	250	99
Refugio	49.4%	21.6%	0.0	17	25	240
Roberts	22.5%	15.5%	0.0	225	133	240
Robertson	45.7%	19.0%	11.2	39	55	159
Rockwall	25.0%	11.6%	17.8	211	218	66
Runnels	46.8%	22.7%	35.0	28	16	12
Rusk	26.9%	13.5%	14.5	196	183	100
Sabine	14.6%	10.1%	30.3	249	236	17
San Augustine	24.4%	11.7%	18.3	214	214	62
San Jacinto	30.3%	14.2%	25.0	163	168	26
San Patricio	46.6%	17.7%	16.9	29	85	79
San Saba	40.2%	22.8%	8.7	74	15	190
Schleicher	30.6%	13.6%	13.9	158	179	111
Scurry	45.8%	26.3%	5.9	36	8	214

© 2024 Adekanmbi V et al. *JAMA Network Open.*

Shackelford	36.9%	15.4%	45.9	109	136	6
Shelby	18.0%	9.2%	7.7	240	240	195
Sherman	22.8%	9.0%	0.0	220	242	240
Smith	36.6%	18.2%	14.4	111	71	102
Somervell	28.5%	13.6%	16.3	180	181	86
Starr	29.1%	12.7%	5.9	174	196	212.5
Stephens	37.0%	18.7%	17.4	105	62	72
Sterling	35.2%	20.5%	62.5	123	33	2
Stonewall	38.0%	14.1%	64.9	95	169	1
Sutton	36.9%	18.4%	0.0	110	66	240
Swisher	40.0%	20.7%	9.5	76	32	183
Tarrant	34.3%	17.5%	15.1	130	91	93
Taylor	38.8%	17.2%	16.3	85	99	85
Terrell	15.3%	8.5%	35.5	247	243	11
Terry	35.4%	18.3%	9.0	119	70	186
Throckmorton	37.0%	14.0%	16.0	106	172	88
Titus	48.2%	18.0%	10.3	22	79	171
Tom Green	46.2%	16.9%	13.7	32	106	118
Travis	39.7%	17.2%	13.6	79	102	119
Trinity	42.4%	21.1%	24.2	58	31	28
Tyler	27.7%	14.4%	24.7	187	163	27
Upshur	28.4%	14.0%	20.7	181	171	41
Upton	42.8%	27.0%	0.0	55	6	240
Uvalde	39.5%	19.2%	14.2	82	49	104
Val Verde	27.0%	14.4%	7.1	195	165	202
Van Zandt	29.9%	13.8%	15.1	166	176	94
Victoria	51.4%	17.2%	16.5	10	101	84
Walker	29.8%	15.2%	22.6	167	140	34
Waller	38.7%	18.1%	16.6	88	76	83
Ward	21.0%	13.2%	6.8	234	191	205
Washington	42.5%	16.8%	15.0	57	109	95
Webb	37.4%	14.5%	7.5	101	156	197
Wharton	51.0%	21.2%	12.2	12	30	141
Wheeler	22.6%	13.5%	13.7	224	186	116
Wichita	27.6%	11.3%	13.9	190	223	110
Wilbarger	33.6%	18.6%	3.0	131	63	224
Willacy	53.1%	34.9%	5.1	8	1	217
Williamson	35.4%	15.5%	13.4	118	134	125
Wilson	30.9%	15.2%	7.8	154	139	194
Winkler	25.4%	13.3%	4.6	207	187.5	220
Wise	25.5%	11.7%	11.1	204	215	161
Wood	30.4%	14.7%	16.8	161	149	82
Yoakum	12.6%	6.8%	17.2	251	249	74

© 2024 Adekanmbi V et al. *JAMA Network Open.*

Young	25.0%	15.0%	28.6	210	144	19
Zapata	45.9%	21.6%	7.2	35	26	201
Zavala	42.7%	28.4%	12.7	56	4	137

Spearman correlation coefficient 0.07 0.06

P value 0.25 0.38

Pctini: percentage of clients with at least one dose of HPV vaccine given the demographic grouping.

Pctutd: Percentage of clients that are up to date on their HPV series given their demographic grouping and age of initiation.

Rate: HPV-related cancer incidence rate.

pctini_rank: rank of % initiation.

pctutd_rank: rank of % up-to-date.

rate_rank: rank of cancer incidence rate.

© 2024 Adekanmbi V et al. *JAMA Network Open.*
