# Supplementary material for: Human Papillomavirus Vaccination and Human Papillomavirus–Related Cancer Rates
Source: JAMA Netw Open. 2024 Sep 5;7(9):e2431807. doi: 10.1001/jamanetworkopen.2024.31807 (PMC11378004; doi:10.1001/jamanetworkopen.2024.31807)
Supplement: Supplement 2. — Data Sharing Statement [file jamanetwopen-e2431807-s002.pdf]

Data Sharing Statement

Adekanmbi. Human Papillomavirus Vaccination and Human Papillomavirus–Related Cancer Rates. *JAMA Netw Open*. Published September 05, 2024.
doi:10.1001/jamanetworkopen.2024.31807

Data
Data available: No

Additional Information
Explanation for why data not available: The data that support the findings of our study are available from the corresponding author upon reasonable request and with permission of the Texas Department of State Health Services.
